# Persistent Systemic Inflammation Mediates the Impact of Postoperative Complications on Survival After Gastric Cancer Surgery

**DOI:** 10.1245/s10434-026-19241-9

**Published:** 2026-02-16

**Authors:** Jane Chungyoon Kim, Hyuk-Joon Lee, Min Kyu Kang, Kyoyoung Park, Sa-Hong Kim, Jeesun Kim, Sung Min Kim, Yo-Seok Cho, Dong-Seok Han, Hye Seong Ahn, Seong-Ho Kong, Do Joong Park, Han-Kwang Yang

**Affiliations:** 1 Department of Surgery, Seoul National University Hospital, Seoul, South Korea; 2 Department of Surgery and Cancer Research Institute, Seoul National University College of Medicine, Seoul, South Korea; 3 Department of Transdisciplinary Medicine, Seoul National University Hospital, Seoul, South Korea; 4 Department of Surgery, Seoul National University Boramae Hospital, Seoul National University College of Medicine, Seoul, South Korea

**Keywords:** Gastric cancer, Neutrophil-to-lymphocyte ratio, Postoperative complications, Systemic inflammation, Survival

## Abstract

**Background:**

Postoperative complications (POCs) are associated with poor long-term outcomes in gastric cancer. However, the underlying biologic mechanisms remain unclear, and systemic inflammation may mediate this association.

**Methods:**

This study retrospectively analyzed 4177 gastric cancer patients who underwent curative gastrectomy between 2013 and 2018. The neutrophil-to-lymphocyte ratio (NLR) was assessed preoperatively (pre-NLR), on postoperative day 2 (early NLR), and 3 months postoperatively (late NLR). Multivariable Cox and logistic regression models assessed the associations of NLR and POCs with survival and complication risk.

**Results:**

For 20.3% of the patients, POCs occurred and were associated with significantly higher NLR at all time points. In the multivariable analysis, early and late NLR independently predicted complication risk. In the Kaplan–Meier survival analysis, POCs were significantly associated with worse overall survival (OS) (hazard ratio [HR], 1.80; *p* < 0.001) and disease-specific survival (DSS) (HR, 1.80; *p* < 0.001). However, in multivariate COX regression, POCs were not independently associated with OS or DSS, whereas late NLR remained a significant predictor for both OS (HR, 1.02; *p* = 0.049) and DSS (HR, 1.03; *p* = 0.008). When stratified by POC status, high late NLR was significantly associated with worse OS (HR, 2.776; *p* < 0.001) and DSS (HR, 2.677; *p* < 0.001) in the POC group. In the no-POC group, high late NLR also was associated with worse OS (HR, 1.587; *p* < 0.001), but not DSS (HR, 1.274; *p* = 0.150).

**Conclusions:**

Persistent systemic inflammation, reflected by elevated late NLR, is a key mediator of the relationship between POCs and survival. Monitoring persistent inflammation may improve risk stratification and guide management strategies for gastric cancer patients.

**Supplementary Information:**

The online version contains supplementary material available at 10.1245/s10434-026-19241-9.

Gastric cancer remains a challenging problem even with recent developments in therapeutic approaches. Still, the curative treatment for gastric cancer remains gastrectomy with regional lymph node dissection (LND).^1^ Depending on the surgical method, extent of LND, and cancer stages, the rates of postoperative complications (POCs) after gastric cancer surgery range from 16 to 44%.^2–4^ As reported by previous studies, POCs compromise long-term survival in diverse malignancies, including esophageal,^5,6^ colorectal,^7,8^ pancreatic,^9,10^ and lung^11,12^ cancers. Similarly, POCs are associated with a poor prognosis for gastric cancer patients.^13–15^

Although numerous studies have linked POCs to negative survival outcomes, the exact mechanisms behind this relationship remain obscure. Some research has suggested that poor survival is associated with complication grade, measured by the Clavien-Dindo (C-D) classification,^16,17^ whereas in other researches, a more reliable predictor has been proposed as the Comprehensive Complication Index, the sum of all complication burdens.^18,19^ Other studies have suggested that failure to complete multimodal treatment due to postoperative morbidities caused worse survival outcomes.^20,21^ On the other hand, infectious complications have received significant interest, with many researchers hypothesizing that inflammations promote tumor growth by modifying the tumor microenvironment to enhance cancer cell proliferation.^22–24^ These various points of view demonstrate the complicated nature of the interaction between POCs and survival, highlighting the need for biologic markers that can resolve this clinical gap.

Among candidate biomarkers, the neutrophil-to-lymphocyte ratio (NLR) has emerged as a simple and dynamic marker of systemic inflammation. Higher NLR indicates a relative increase in neutrophil-mediated tumor-promoting activity and a decline in lymphocyte-mediated anti-tumor immunity.^25^ Although preoperative NLR has been suggested as a prognostic indicator in gastric cancer,^26,27^ its usefulness in understanding the biologic effects of postoperative complications has not been investigated. In particular, little is known about how NLR changes over time and whether chronic inflammation, indicated by higher NLR in the postoperative phase, affects the long-term prognostic impact of complications.

This study explored the dynamic role of NLR in the relationship between POCs and survival among gastric cancer patients who received gastrectomy. By analyzing NLR levels at three time points (preoperatively, on postoperative day 2, and 3 months after surgery), we sought to explain whether persistent cancer-related inflammation affects long-term results and changes the prognostic significance of surgical complications.

## Methods

### Patients

This retrospective cohort study used a prospectively maintained complication database at the Department of Surgery, Seoul National University Hospital. The study enrolled patients with biopsy-proven gastric adenocarcinoma who underwent curative gastrectomy from January 2013 to December 2018. Gastrectomy and LND procedures were performed in accordance with the Korean Gastric Cancer Association guidelines.^1^ For clinically advanced cancer stages, we performed either distal or total gastrectomy with D2 LND. For early cancer stages, we chose distal, total, pylorus-preserving, or proximal gastrectomy according to the tumor location or size and performed either D1 or D1 plus LND. Neoadjuvant chemotherapy was not routinely performed, and nearly all patients underwent upfront curative gastrectomy followed by adjuvant chemotherapy when indicated.

Postoperative care during the study period followed conventional perioperative care. Oral intake was initiated with sips of water on postoperative day 3, followed by a soft fluid diet on postoperative day 4 and a soft-blend diet on postoperative day 6, with discharge typically around postoperative day 7. Other aspects of perioperative care, including anesthesia, fluid management, and postoperative analgesia, were applied according to institutional standards and were consistent across the cohort.

The exclusion criteria ruled out (1) emergency operations due to cancer perforation or intractable bleeding and (2) non-curative resection or non-resection procedures such as palliative gastrojejunostomy. Pathologic staging was performed based on the eighth edition of the American Joint Committee on Cancer (AJCC) staging system for gastric cancer.^28^ The research received approval from the Institutional Review Board of Seoul National University Hospital (no. 2311-072-1483).

### Postoperative Complications Data

All POCs were recorded and classified according to the C-D classification system.^29^ Our data collection methods for POCs have been detailed in previous studies.^30,31^ The attending surgeons prospectively recorded daily clinical courses and events. Weekly meetings with all faculty members were held to reach a consensus on C-D grading, ensuring consistency and inter-surgeon agreement in complication grading.

Complications within the first 30 days after surgery were recorded irrespective of whether the patient was re-admitted or revisited. Complications detected after the initial 30-day postoperative period were considered if they occurred during the same hospitalization period. Specific types of complication also were recorded in detail, including those related to surgical-site infections, infections in other areas, and medical problems.

### Data Collection

Clinicopathologic and surgical information was extracted from a prospectively collected gastric cancer database. Variables included age, sex, type of gastrectomy, surgical approach, extent of LND, type of POC, and C-D grade. We merged our database with hospital-wide patient databases to obtain additional information, including preoperative body mass index (BMI), medical history, comorbidities, completion of adjuvant therapy, and laboratory results. Baseline serum albumin levels measured preoperatively and American Society of Anesthesiologists (ASA) physical status classification assessed at the time of anesthesia evaluation also were extracted. The Charlson Comorbidity Index (CCI) was calculated using the medical history documented on the day of admission for surgery.^32^

The study assessed NLR at three time points: preoperatively (pre-NLR), on postoperative day 2 (early NLR), and 3 months postoperatively (late NLR), representing baseline inflammation, acute postoperative response, and persistent inflammation, respectively. Pre-NLR was obtained from routine laboratory tests performed at the time of outpatient surgical evaluation, typically 2–8 weeks before surgery. All patients were clinically stable and free of active infection or systemic inflammatory conditions at the time of blood sampling.

For long-term prognostic analysis, overall survival (OS) and disease-specific survival (DSS) data were obtained from the Korean National Health Information Database (NHID). The study measured DSS as the interval from the date of surgery to the date of death specifically caused by gastric cancer.

### Statistical Analysis

Categorical variables were evaluated using Pearson’s chi-square test, and continuous variables were compared using the Student’s *t* test. The Kaplan-Meier method was used to generate survival curves, and the log-rank (Mantel-Cox) test was used to assess the disparities between the curves. Cox proportional hazard models assessed the impact of POCs on OS and DSS, controlling for factors including age, sex, BMI, CCI score, pathologic stage, surgical type, LND extent, and adjuvant therapy.

Interaction and stratified analyses were performed to explore the modifying effect of NLR levels on the relationship between complications and survival outcomes. All statistical analyses were performed using R (version 4.4.1), and survival graphs were generated with GraphPad Prism (version 10.2.3).

## Results

### Study Population

The analysis included 4177 patients who underwent gastric cancer surgery. Of these patients, 850 (20.3%) experienced POCs, including 420 patients (10.1%) with minor POCs (C-D grade 1 or 2) and 430 patients (10.3%) with major POCs (C-D grade ≥ 3) (Table 1). Male gender, old age, high CCI high ASA, and advanced tumor stage all were strongly correlated with POCs (all *p* < 0.001). Serum albumin levels did not differ significantly between the patients with and without POCs (4.15 ± 0.35 vs. 4.14 ± 0.35; *p* = 0.374). In addition, strong correlations were found between the POCs and surgical characteristics, such as total gastrectomy, open surgery, and D2 LND (all *p* < 0.001).

**Table 1:** Clinicopathologic and operative characteristics according to postoperative complication grade (total *n* = 4177)

Characteristic	No complications (*n* = 3327) *n* (%)	All complications (*n* = 850) *n* (%)	*p* value	C-D grade ≥ 3 (*n* = 430) *n* (%)	*p* value
Sex					
Male	2055 (76.9)	617 (23.1)	< 0.001	312 (11.7)	< 0.001
Female	1272 (84.5)	233 (15.5)		118 (7.8)	
Age (years)					
–50	669 (84.3)	125 (15.7)	< 0.001	62 (7.8)	< 0.001
51–60	947 (80.9)	223 (19.1)		120 (10.3)	
61–70	954 (78.8)	257 (21.2)		133 (11)	
≥ 71	757 (75.5)	245 (24.5)		115 (11.5)	
BMI					
–18.5	91 (76.5)	28 (23.5)	0.676	15 (12.6)	0.547
18.5–25	1985 (79.8)	502 (20.2)		261 (10.5)	
≥ 25	1251 (79.6)	320 (20.4)		154 (9.8)	
CCI					
2	506 (83.6)	99 (16.4)	< 0.001	52 (8.6)	< 0.001
3–4	1679 (81.5)	380 (18.5)		199 (9.7)	
≥ 5	1142 (75.5)	371 (24.5)		179 (11.8)	
ASA score	1.66 ± 0.54	1.77 ± 0.58	< 0.001	1.77 ± 0.60	< 0.001
Disease stage					
I	2434 (81.8)	540 (18.2)	< 0.001	270 (9.1)	< 0.001
II	451 (79.3)	118 (20.7)		63 (11.1)	
III	442 (69.7)	192 (30.3)		97 (15.3)	
Operation type					
DG	1988 (81.4)	454 (18.6)	< 0.001	221 (9)	< 0.001
TG	607 (71.8)	238 (28.2)		117 (13.8)	
PPG	648 (82.8)	135 (17.2)		80 (10.2)	
PG	84 (78.5)	23 (21.5)		12 (11.2)	
Approach					
Open	721 (70.6)	300 (29.4)	< 0.001	147 (14.4)	< 0.001
Laparoscopy	2342 (82.7)	489 (17.3)		243 (8.6)	
Robot	264 (81.2)	61 (18.8)		40 (12.3)	
Lymphadenectomy					
Under D2	2112 (82.1)	462 (17.9)	< 0.001	232 (9)	< 0.001
D2	1215 (75.8)	388 (24.2)		198 (12.4)	
R0 resection	3325 (99.9)	849 (99.9)	0.571	429 (99.9)	0.226
Resected LNs	40.59 ± 15.91	41.44 ± 16.50	0.168	40.08 ± 15.79	0.359
Adjuvant therapy (stage II/III)					
Complete	626 (75.2)	206 (24.8)	0.231	103 (12.4)	0.159
Incomplete	267 (72.0)	104 (28.0)		57 (15.4)	
Albumin	4.15 ± 0.35	4.14± 0.35	0.374	4.13 ± 0.35	0.078
Pre-NLR	2.03 ± 1.23	2.18 ± 1.36	0.005	2.20 ± 1.43	0.010
Early NLR	8.52 ± 4.84	10.25 ± 7.35	< 0.001	10.42 ± 7.95	< 0.001
Late NLR	1.57 ± 1.27	1.91 ± 4.22	0.016	2.09 ± 5.53	0.038

C-D, Clavien-Dindo; BMI, body mass index; CCI Charlson Comorbidity Index; ASA, American Association of Anesthesiologists; DG, distal gastrectomy; TG, total gastrectomy; PPG, pylorus-preserving gastrectomy; PG, proximal gastrectomy, LN, lymph node; NLR, neutrophil-to-lymphocyte ratio

The mean number of retrieved lymph nodes was comparable between the patients with and without POCs, averaging 40–41 nodes, and the R0 resection rate was uniformly high, at 99.9% (3325/3327 in the POC group and 849/850 in the no-POC group). In advanced stages, a higher proportion of patients failed to complete adjuvant therapy if they had POCs, but the difference was not significant (*p* = 0.231).

The NLR levels at all three time points were significantly higher in the POC group than in the no-POC group. The mean pre-NLR was 2.18 ± 1.36 in the POC group versus 2.03 ± 1.23 in the no-POC group (*p* = 0.005). Early NLR also was significantly elevated in the POC group (10.25 ± 7.35 vs. 8.52 ± 4.84; *p* < 0.001). Similarly, late NLR was higher among the patients with POCs (1.91 ± 4.22 vs. 1.57 ± 1.27; *p* = 0.016).

Detailed types and grades of POCs are summarized in Table S1. Both surgical and medical events were recorded. The most common type was intra-abdominal fluid collection or abscess, which occurred in 4.7% of the patients, including 2.6% requiring percutaneous drainage (C-D grade ≥ 3). Other frequent events included superficial wound problems (2.6%), anastomotic stenosis (2.4%), and pulmonary complications (3.5%).

To further characterize the surgical background, surgical and inflammatory outcomes according to surgery type are shown in Table S2. The overall complication rate was higher after total gastrectomy (28.2%) than after other procedures including distal (18.6%), pylorus-preserving (17.2%), and proximal gastrectomy (21.5%; *p* < 0.001). The 30-day mortality remained 0.1% across all procedure types. Pre-NLR and early NLR were significantly higher in the total gastrectomy group, whereas late-NLR values did not differ significantly among procedures. In addition, to evaluate the impact of postoperative complications on adjuvant chemotherapy, detailed treatment outcomes, including completion status, regimen, delay, and dose reduction, are summarized in Table 2. Treatment delay (> 8 weeks) occurred more frequently for patients with complications (*p* < 0.001), whereas completion and dose-reduction rates were comparable between groups.

**Table 2:** Adjuvant chemotherapy treatment outcomes for stage II/III patients (*n* = 1203)^a^

Outcome	No complication *n* (%)	Complication* n* (%)	*p* value
Adjuvant chemotherapy			
Complete	626 (75.2)	206 (24.8)	0.231
Incomplete/not started	267 (72.0)	104 (28.0)	
Regimen			
XELOX	490 (75.5)	159 (24.5)	0.950
TS-1	287 (75.3)	94 (24.7)	
Delay			
No delay	738 (77.4)	216 (22.6)	< 0.001
Delay (> 8 weeks)	39 (51.3)	37 (48.7)	
Dose reduction			
No dose reduction	570 (75.6)	184 (24.4)	0.844
Dose reduction	207 (75.0)	69 (25.0)	

^a^Analyses of regimen, delay, and dose reduction were restricted to patients who initiated adjuvant chemotherapy; those who never started were excluded from these denominators.

### Long-Term Survival Analysis

**Fig. 1:**
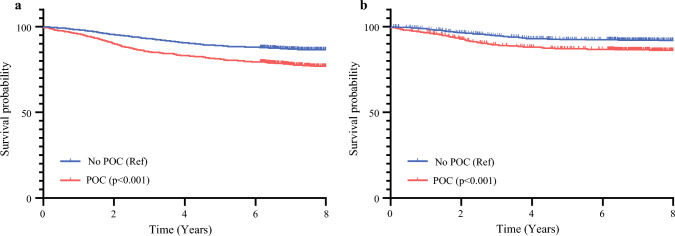
Survival curves comparing patients with and without postoperative complications (POCs). **a** Overall survival (OS). **b** Disease-specific survival (DSS)

The Kaplan–Meier survival analysis showed that POCs were significantly associated with reduced OS compared with the no-POC cohort (hazard ratio [HR], 1.801; 95% CI 1.521–2132; *p* < 0.001), as determined by the log-rank test (Fig. 1a). The DSS analysis also indicated that POCs were associated with a markedly poorer outcome compared with the no-POC group (HR, 1.803; 95% CI 1.443–2.253; *p* < 0.001; Fig. 1b).

In subgroup analyses by pathologic stage, the negative impact of POCs on survival was evident across all stages. In stage I, cancer patients with POCs showed a significantly lower OS (HR, 2.112; 95% CI 1.597–2.794; *p* < 0.001) and DSS (HR, 1.915; 95% 1.070–3.428; *p* = 0.029). In advanced cancer stages (II–III), POCs still were associated with poorer OS (HR, 1.311; 95% 1.061–1.619; *p* = 0.012) and DSS (HR, 1.360; 95% CI 1.069–1.731; *p* = 0.012).

In univariate analysis, multiple variables were significantly associated with both OS and DSS, including sex, age, BMI, CCI cancer stage, surgery type, surgical approach, extent of LND, completion of adjuvant therapy, POCs, and NLR, at all three time points (Table 3). However, in multivariate analysis, POCs were not identified as an independent prognostic factor for either OS (HR, 1.174; 95% CI 0.924–1.492; *p* = 0.190) or DSS (HR, 1.158; 95% CI 0.881–1.524; *p* = 0.293). Pre-NLR remained a significant factor for OS (HR, 1.069; 95% CI 1.008–1.133; *p* = 0.026), but did not reach statistical significance for DSS (HR, 1.068; 95% CI 0.995–1.145; *p* = 0.067). Early NLR was not associated with either OS (HR, 1.003; 95% CI 0.988–1.018; *p* = 0.711) or DSS (HR, 1.008; 95% CI 0.992–1.023; *p* = 0.331). In contrast, late NLR was an independent prognostic factor for both OS (HR, 1.019; 95% CI 1.002–1.038; *p* = 0.049) and DSS (HR, 1.025; 95% CI 1.007–1.043; *p* = 0.008). Other independent predictors of OS included BMI, cancer stage, surgical approach, and completion of adjuvant therapy. For DSS, only cancer stage and adjuvant therapy remained significant.

**Table 3:** Uni- and multivariate analyses of overall survival (OS) and disease-specific survival (DSS) according to postoperative complications and associated factors

Factors	OS univariate HR (95% CI)	*p* value	OS multivariate HR (95% CI)	*p* value	DSS univariate HR (95% CI)	*p* value	DSS multivariate HR (95% CI)	*p* value
Male sex	1.321 (1.117–1.562)	0.001	1.018 (0.810–1.280)	0.878	1.033 (0.835–1.279)	0.763	0.898 (0.694–1.162)	0.898
Age	1.048 (1.040–1.056)	< 0.001	1.006 (0.988–1.024)	0.518	1.022 (1.013–1.032)	< 0.001	1.001 (0.981–1.022)	0.906
BMI	0.917 (0.894–0.941)	< 0.001	0.950 (0.918–0.983)	0.003	0.924 (0.894–0.956)	< 0.001	0.962 (0.926–1.000)	0.051
CCI	1.487 (1.410–1.568)	< 0.001	1.071 (0.917–1.251)	0.385	1.208 (1.123–1.299)	< 0.001	1.023 (0.851–1.230)	0.808
Disease stage								
I	Ref	Ref	Ref	Ref	Ref	Ref	Ref	Ref
II	2.902 (2.326–3.621)	< 0.001	1.801 (1.370–2.367)	< 0.001	6.458 (4.504–9.259)	< 0.001	4.278 (2.762–6.626)	< 0.001
III	7.946 (6.684–9.446)	< 0.001	4.713 (3.661–6.067)	< 0.001	26.900 (20.077–36.041)	< 0.001	15.960 (10.676–23.859)	< 0.001
Operation type								
DG	Ref	Ref	Ref	Ref	Ref	Ref	Ref	Ref
TG	1.806 (1.53–2.132)	< 0.001	1.224 (0.975–1.536)	0.081	2.280 (1.849–2.811)	< 0.001	1.219 (0.941–1.578)	0.133
PPG	0.260 (0.183–0.37)	< 0.001	0.825 (0.322–2.114)	0.689	0.086 (0.038–0.194)	< 0.001	0.675 (0.201–2.270)	0.675
PG	0.683 (0.375–1.244)	0.212	–	–	0.332 (0.106–1.039)	0.058	–	–
Approach								
Open	Ref	Ref	Ref	Ref	Ref	Ref	Ref	Ref
MIS	0.240 (0.206–0.208)	< 0.001	0.768 (0.593–0.994)	0.045	0.155 (0.125–0.192)	< 0.001	0.775 (0.575–1.044)	0.093
LND								
< D2	Ref	Ref	Ref	Ref	Ref	Ref	Ref	Ref
D2	3.456 (2.935–4.069)	< 0.001	1.161 (0.855–1.575)	0.340	6.187 (4.834–7.918)	< 0.001	1.142 (0.803–1.623)	0.460
Adjuvant therapy								
Complete	Ref	Ref	Ref	Ref	Ref	Ref	Ref	Ref
Incomplete	2.517 (2.075–3.054)	< 0.001	2.329 (1.846–2.939)	< 0.001	2.054 (1.640–2.571)	< 0.001	2.062 (1.575–2.700)	< 0.001
Complication								
No	Ref	Ref	Ref	Ref	Ref	Ref	Ref	Ref
Yes	1.801 (1.521–2.132)	< 0.001	1.174 (0.924–1.492)	0.190	1.800 (1.440–2.249)	< 0.001	1.158 (0.881–1.524)	0.293
Pre-NLR	1.167 (1.131–1.204)	< 0.001	1.069 (1.008–1.133)	0.026	1.168 (1.124–1.217)	< 0.001	1.068 (0.995–1.145)	0.067
Early NLR	1.029 (1.021–1.037)	< 0.001	1.003 (0.988–1.018)	0.711	1.033 (1.024–1.042)	< 0.001	1.008 (0.992–1.023)	0.331
Late NLR	1.030 (1.016–1.044)	< 0.001	1.019 (1.002–1.038)	0.049	1.031 (1.014–1.048)	< 0.001	1.025 (1.007–1.043)	0.008

OS, overall survival; HR, hazard ratio; CI confidence interval; CCI Charlson Comorbidity Index; NLR, neutrophil-to-lymphocyte ratio; DG, distal gastrectomy; TG, total gastrectomy; PPG, pylorus-preserving gastrectomy; PG, proximal gastrectomy; MIS, minimally invasive surgery; LND, lymphadenectomy; NLR, neutrophil-to-lymphocyte ratio

### Factors Contributing to Postoperative Complication

In univariate analysis, several factors were associated with the occurrence of POCs. Male sex, old age, high CCI elevated NLR at all three time points, stage III cancer, total gastrectomy, open surgery, and D2 LND were linked to high POC rates. Completion of adjuvant therapy was not associated with the POCs (Table 4).

**Table 4:** Uni- and multivariate analyses of factors associated with postoperative complications

Factors	Univariate OR (95% CI)	*p* value	Multivariate OR (95% CI)	*p* value
Male sex	1.639 (1.388–1.935)	< 0.001	1.587 (1.149–2.192)	0.005
Age	1.016 (1.010–1.023)	< 0.001	0.985 (0.963–1.008)	0.208
BMI	1.003 (0.980–1.026)	0.815	1.031 (0.987–1.076)	0.176
CCI	1.178 (1.116–1.243)	< 0.001	1.255 (1.028–1.532)	0.026
Pre-NLR	1.086 (1.028–1.148)	0.003	0.967 (0.877–1.067)	0.508
Early NLR	1.053 (1.038–1.069)	< 0.001	1.035 (1.011–1.059)	0.003
Late NLR	1.068 (1.023–1.116)	0.003	1.075 (1.005–1.150)	0.035
Disease stage				
I	Ref	Ref	Ref	Ref
II	1.179 (0.944–1.474)	0.147	0.977 (0.747–1.278)	0.867
III	1.958 (1.614–2.376)	< 0.001	1.361 (1.033–1.793)	0.029
Operation type				
DG	Ref	Ref	Ref	Ref
TG	1.717 (1.432–2.058)	< 0.001	1.276 (0.934–1.744)	0.126
PPG	0.912 (0.738–1.127)	0.395	1.003 (0.368–2.731)	0.996
PG	1.199 (0.748–1.923)	0.452	1.214 (0.134–11.013)	0.863
Approach				
Open	Ref	Ref	Ref	Ref
MIS	0.507 (0.431–0.597)	< 0.001	0.76 (0.543–1.064)	0.110
LND				
< D2	Ref	Ref	Ref	Ref
D2	1.460 (1.254–1.700)	< 0.001	0.957 (0.683–1.341)	0.423
Adjuvant therapy				
Complete	Ref	Ref	Ref	Ref
Incomplete	1.187 (0.901–1.562)	0.222	0.957 (0.683–1.341)	0.797

OR, odds ratio; CI confidence interval; BMI, body mass index; CCI Charlson Comorbidity Index; NLR, neutrophil-to-lymphocyte ratio; DG, distal gastrectomy; TG, total gastrectomy; PPG, pylorus-preserving gastrectomy; PG, proximal gastrectomy; MIS, minimally invasive surgery; LND, lymphadenectomy

In multivariate analysis, only male sex, high CCI early NLR, late NLR, and stage III cancer remained independently associated with POCs. Early NLR showed an odds ratio (OR) of 1.035 (95% CI 1.011–1.059; *p* = 0.003) for the development of POCs, whereas late NLR had an OR of 1.075 (95% CI 1.005–1.150; *p* = 0.035). Pre-NLR was not significantly associated (OR, 0.967; 95% CI 0.877–1.067; *p* = 0.508).

### Survival Analyses Comparing High and Low Late NLR

Among the three types of NLR, only late NLR was independently associated with both POCs and survival. Therefore, we hypothesized that late NLR may mediate the impact of POCs on survival. To determine the optimal cutoff value for late NLR, receiver operating characteristic (ROC) curve analysis was performed using OS as the outcome variable. Based on this analysis, the area under the curve (AUC) was 0.680, and a threshold of 2.18 was identified as the optimal cutoff point. Patients were stratified into low (≤ 2.18) and high (> 2.18) late-NLR groups.

Multivariate survival analyses then were performed separately within each group. For OS, covariates previously identified as significant (BMI, cancer stage, surgical approach, LND extent, and adjuvant therapy) were included, together with presence of POCs. For DSS, only cancer stage, adjuvant therapy, and POCs were included as covariates (Table 5).

**Table 5:** Survival analysis comparing low (≤ 2.18) and high (> 2.18) late NLR groups

Factors	OS				DSS			
	Multivariate				Multivariate			
	Low NLR HR (95% CI)	*p* value	High NLR HR (95% CI)	*p* value	Low NLR HR (95% CI)	*p* value	High NLR HR (95% CI)	*p* value
Age	–	–	–	–	–	–	–	–
BMI	0.975 (0.938–1.014)	0.473	0.904 (0.853–0.959)	< 0.001	–	–	–	–
CCI	–	–	–	–	–	–	–	–
Disease stage								
I	Ref	Ref	Ref	Ref	Ref	Ref	Ref	Ref
II	1.982 (1.415–2.778)	< 0.001	1.754 (1.142–2.695)	0.010	4.810 (2.830–8.177)	< 0.001	3.805 (1.867–7.758)	< 0.001
III	5.435 (3.979–7.422)	< 0.001	4.277 (2.883–6.345)	< 0.001	17.188 (10.475–28.203)	< 0.001	14.898 (7.864–28.223)	< 0.001
Approach								
Open	Ref	Ref	Ref	Ref	–	–	–	–
MIS	0.743 (0.547–1.008)	0.056	0.788 (0.518–1.198)	0.264	–	–	–	–
LND								
< D2	Ref	Ref	Ref	Ref	–	–	–	–
D2	1.133 (0.807–1.591)	0.471	1.263 (0.734–2.173)	0.398	–	–	–	–
Complication								
No	Ref	Ref	Ref	Ref	Ref	Ref	Ref	Ref
Yes	0.887 (0.664–1.187)	0.420	1.612 (1.097–2.368)	0.015	0.930 (0.668–1.294)	0.666	1.576 (1.007–2.466)	0.047
Adjuvant therapy								
Complete	Ref	Ref	Ref	Ref	Ref	Ref	Ref	Ref
Incomplete	2.051 (1.549–2.716)	< 0.001	3.453 (2.319–5.142)	< 0.001	1.740 (1.247–2.427)	0.001	2.760 (1.746–4.363)	< 0.001

OS, overall survival; DSS, disease-specific survival; NLR, neutrophil-to-lymphocyte ratio; HR, hazard ratio; CI confidence interval; BMI, body mass index; CCI Charlson Comorbidity Index; MIS, minimally invasive surgery; LND, lymphadenectomy

In the low late-NLR group, POCs were not significantly associated with either OS (HR, 0.887; 95% CI 0.664–1.187; *p* = 0.420) or DSS (HR, 0.930; 95% CI 0.668–1.254; *p* = 0.666). In contrast, in the high-NLR group, POCs were identified as independent prognostic factors for both OS (HR, 1.612; 95% CI 1.097–2.368; *p* = 0.015) and DSS (HR, 1.576; 95% CI 1.007–2.466; *p* = 0.047).

**Fig. 2:**
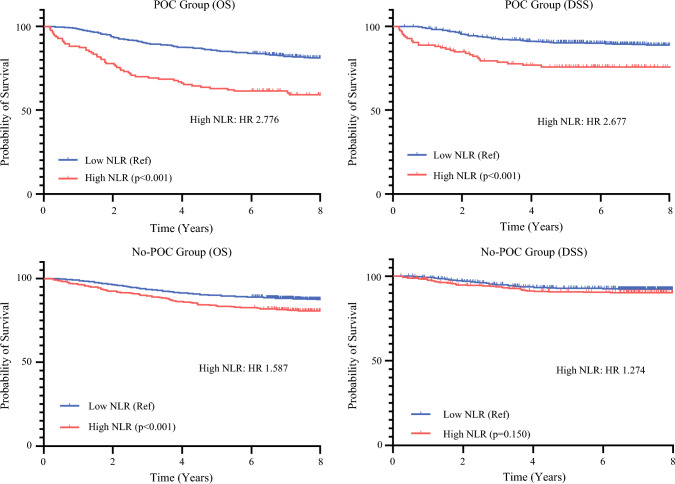
Kaplan–Meier curves comparing overall survival (OS) and disease-specific survival (DSS) stratified by postoperative complications (POCs) and late neutrophil-to-lymphocyte ratio (NLR) levels (high vs. low)

We performed Kaplan–Meier survival analyses comparing patients with high versus low late-NLR levels (Fig. 2). In the POC group, high late NLR was significantly associated with worse OS (HR, 2.776; 95% CI 2.001–3.867; *p* < 0.001) and DSS (HR, 2.677; 95% CI 1.503–4.769; *p* < 0.001). In the no-POC group, high late NLR also was linked to poorer OS (HR, 1.587; 95% CI 1.252–2.011; *p* < 0.001), although the effect size was smaller than in the POC group. For DSS, however, the difference between the high and low late-NLR groups was not statistically significant (HR, 1.274; 95% CI 0.890–1.823; *p* = 0.150). In addition, the 5 year-DSS was 76.0% (95% CI 68.4–83.6%) for the patients with both POCs and high late NLR, which was significantly lower than in the other groups: 92.7% (95% CI 91.7–93.7%) in the no-POC/low late-NLR group, 90.8% (95% CI 88.1–93.5%) in the no-POC/high late-NLR group, and 90.2% (95% CI 87.8–92.6%) in the POC/low late-NLR group.

## Discussion

This study demonstrated a complex relationship between POCs, systemic inflammation, and survival after curative gastric cancer surgery. In univariate analyses, POCs were significantly associated with worse OS and DSS. However, multivariate analyses with adjustment for clinicopathologic factors showed that POCs were not independent predictors of survival. Measurement of inflammatory markers across different time points showed that pre-NLR and late NLR were independently associated with survival, whereas early NLR and late NLR were independently associated with POCs. Late NLR was the only factor that was associated independently with POCs and survival, suggesting that it may serve as a key mediator of the impact of complications on survival. Notably, the prognostic significance of late NLR remained independent even after adjustment for adjuvant chemotherapy completion, suggesting that the adverse effect of sustained systemic inflammation extends beyond treatment adherence. Furthermore, stratified analyses showed that the adverse impact of high late NLR on survival was evident mostly among patients with POCs, whereas its effect was less prominent among those without POCs. These findings suggest that persistent systemic inflammation mediates the relationship between POCs and long-term oncologic outcomes.

The prognostic roles of NLRs vary by perioperative timing. Pre-NLR reflects the baseline host inflammatory status before surgery and has been associated with survival outcomes but not with the occurrence of POCs. Early NLR, which represents the acute inflammatory response triggered by surgical stress and tissue injury, was significantly associated with the POCs but did not independently predict survival. In contrast, late NLR, assessed 3 months after surgery, captured the sustained systemic inflammatory state. Although acute inflammation may be important for immediate postoperative morbidity, it is the persistence of systemic inflammation over time that ultimately influences survival.

Although previous studies have linked POCs to poor outcomes, their prognostic impact appears to vary depending on clinical and pathologic factors. Some studies have reported a greater effect of complications on patients with advanced-stage cancer. Kubota et al.^13^ showed that as the pathologic stage increased, the impact of complications on the OS and DSS also was amplified. Jung et al.^33^ also reported that complications affected the OS but not the DSS of stage I patients, whereas both the OS and DSS decreased for stage III patients.

Other studies have focused on the severity of complications as an important predictor of survival. Li et al.^16^ demonstrated that higher C-D grades were significantly associated with poor OS and DSS across all stages. Postoperative morbidity also was linked to failure to complete multimodal therapy, which contributed to worse survival after gastrectomy. In the previous study conducted by Li et al.^16^, patients with complications failed to complete all the intended multimodal therapies due to increased morbidities, which resulted in worse 3-year OS rates.^20^ However, in our cohort, the majority of advanced-stage patients completed adjuvant therapy, likely due to timely recovery. Consequently, the negative impact of POCs was not amplified in advanced disease stages, but rather appeared more pronounced in early-stage patients with an otherwise favorable prognosis.

In addition to these factors, some studies have focused on the type of complication itself, particularly the types involving infection. These complications were significantly linked to an increased risk of gastric cancer recurrence and reduced OS compared with patients who had no complications.^24,34^ Furthermore, several studies have shown that intra-abdominal infections were significantly correlated with worse OS and relapse-free survival.^17,22,35^ Other studies have emphasized the prognostic role of postoperative inflammatory markers, especially C-reactive protein (CRP). Kurokawa et al.^23^ reported that elevated CRP levels in the early postoperative period predict not only the development of complications, but also worse oncologic outcomes. These findings suggest that it is not only the presence of complications but the inflammatory burden they create that influences long-term prognosis.

Several previous studies have reported that an elevated preoperative NLR is associated with poor survival in gastric cancer. Yamanaka et al.^36^ performed one of the initial studies showing that preoperative NLR was an independent prognostic factor in advanced gastric cancer. In another large-scale study, Shimada et al.^26^ demonstrated that patients with high NLR (≥ 4.0) had a significantly lower 5-year survival rate than those with low NLR (57 vs. 82%), and that high NLR remained an independent prognostic factor even after adjustment for tumor stage.

A meta-analysis involving 19 studies confirmed that elevated NLR is significantly associated with advanced disease and poor overall and disease-free survival in gastric cancer.^37^ Additionally, Miyamoto et al.^38^ found that elevated preoperative NLR was associated with both poor long-term survival and increased perioperative complications, including bleeding and infection.

Some studies also have reported that postoperative NLR could be a meaningful prognostic indicator. Kim and Song^39^ reported that both elevated preoperative (≥ 2.0) and 6-month postoperative NLR (≥ 1.7) were independently associated with worse OS after gastrectomy. Notably, Min et al.^40^ further emphasized that a persistently elevated NLR 3–6 months after surgery was a stronger predictor of poor survival than preoperative NLR alone. These findings support the conclusion that sustained postoperative inflammation may play a critical role in cancer progression.

The clinical relevance of NLR was first introduced by Zahorec^41^ in 2001, who proposed that surgical stress and critical illness induce a shift toward neutrophilia and lymphocytopenia, reflecting systemic immune dysregulation. This is related to cancer-associated systemic inflammation, which contributes to tumor progression by impairing immune surveillance, promoting angiogenesis, and facilitating metastasis.^25^

Neutrophils in the tumor microenvironment release pro-tumorigenic factors such as vascular endothelial growth factor (VEGF), matrix metalloproteinase-9 (MMP-9), and fibroblast growth factor-2 (FGF-2), whereas lymphocytes exert anti-tumor effects through cytotoxic activity.^42,43^ Our study built on this concept by demonstrating that late NLR captures the persistence of cancer-associated inflammation and significantly modifies the prognostic effect of POCs. These findings suggest that long-term oncologic outcomes are shaped not simply by the occurrence of complications, but by their interaction with unresolved systemic inflammation. Late NLR may serve as a practical biomarker to identify high-risk patients and guide postoperative strategies targeting cancer-associated inflammation.

Although this study provides meaningful insights into the relationship between postoperative complications and long-term survival in gastric cancer, several limitations must be acknowledged. Its retrospective design introduced potential selection bias and relied on the accuracy of medical records. Additionally, as a single-center study, the generalizability of the findings may be limited due to differences in surgical practices, patient characteristics, and postoperative care across institutions. Real-world variables such as treatment adherence, postoperative rehabilitation, nutritional status, and follow-up care also may influence systemic inflammation and survival. Frailty and nutritional parameters were not routinely assessed preoperatively during the study period; therefore, the CCI was used as an objective surrogate of baseline health status. Future multi-center prospective studies are needed to validate these findings and explore the clinical utility of inflammation-based risk stratification.

This study highlights that the long-term impact of postoperative complications depends on the patients’ systemic inflammatory state. A persistently elevated NLR in the late postoperative phase was strongly associated with worse outcomes, suggesting that unresolved cancer-associated systemic inflammation plays a key role in mediating the effect of complication on survival. These findings underscore the need for postoperative monitoring of inflammatory markers and raise the possibility that targeting chronic inflammation could improve oncologic outcomes in gastric cancer.

## Supplementary Information

Below is the link to the electronic supplementary material.

Supplementary file1 (DOCX 24 KB)
