# Supplementary material for: Persistent Systemic Inflammation Mediates the Impact of Postoperative Complications on Survival After Gastric Cancer Surgery
Source: Ann Surg Oncol. 2026 Feb 16;33(5):4702–13. doi: 10.1245/s10434-026-19241-9 (PMC13083312; doi:10.1245/s10434-026-19241-9)
Supplement: Supplementary file 1 — Supplementary file1 (DOCX 24 KB) [file 10434_2026_19241_MOESM1_ESM.docx]

**<Supplementary Tables>**

Supplementary Table 1. Details of Postoperative Complications

| **Complication type** | **Includes** | **Any grade, n (%)** | **C-D ≥3, n (%)** |
| --- | --- | --- | --- |
| Wound-related (superficial) | seroma, hematoma, infection, dehiscence | 110 (2.6) | 66 (1.6) |
| Intra-abdominal complication |  |  |  |
| Intra-abdominal fluid collection/abscess | requiring antibiotics or drainage | 195 (4.7) | 107 (2.6) |
| Intra-abdominal bleeding | postoperative hemoperitoneum | 31 (0.7) | 20 (0.5) |
| Luminal bleeding | anastomotic bleeding, intraluminal bleeding | 26 (0.6) | 7 (0.2) |
| Anastomosis stenosis | anastomotic stricture, often requiring balloon dilatation | 101 (2.4) | 87 (2.1) |
| Motility disorder | Ileus, internal hernia, obstruction | 88 (2.1) | 16 (0.4) |
| Gastrointestinal fistula | anastomotic leak, enterocutaneous fistula | 93 (2.2) | 79 (1.9) |
| Other Fistula | chylous leak | 11 (0.3) | 8 (0.2) |
| Remnant stomach Ischemia | Ischemic change of remnant stomach | 10 (0.2) | 4 (0.1) |
| Medical Complication |  |  |  |
| Pulmonary complications | Pneumonia, pleural effusion | 146 (3.5) | 53 (1.3) |
| Urinary complications | Urinary retention, urinary tract infection | 28 (0.7) | 1 (0) |
| Renal complications | Acute kidney injury | 4 (0.1) | 3 (0.1) |
| Hepatobiliary complications | Cholecystitis, cholangitis, liver dysfunction | 25 (0.6) | 5 (0.1) |
| Gastrointestinal | Nausea, vomiting requiring prolonged antiemetic treatment | 20 (0.5) | 1 (0) |
| Other Infection | Phlebitis, catheter-related infection | 37 (0.9) | 2 (0) |
| Cardiac complications | Arrhythmia, myocardial ischemia | 16 (0.4) | 10 (0.2) |
| Neuropsychic complications | Delirium | 23 (0.6) | 2 (0) |
| Vascular complications | Deep vein thrombosis, pulmonary embolism | 21 (0.5) | 3 (0.1) |
| Endocrine complications | Adrenal insufficiency | 3 (0.1) | 1 (0) |
| Unclassified | Rare or miscellaneous complications | 47 (1.1) | 9 (0.2) |

* C-D = Clavien-Dindo grade

Supplementary Table 2. Surgical Outcomes by Procedure type

| **Outcome** | **DG** | **TG** | **PPG** | **PG** | **P-value** |
| --- | --- | --- | --- | --- | --- |
| Patients, n (%) | 2442 | 845 | 783 | 107 |  |
| Stage, n (%) |  |  |  |  | <0.001 |
| I | 1721 (70.5) | 413 (48.9) | 742 (94.8) | 98 (91.6) |  |
| II | 366 (15.0) | 160 (18.9) | 36 (4.6) | 7 (6.5) |  |
| III | 355 (14.5) | 272 (32.2) | 5 (0.6) | 2 (1.9) |  |
| Any Complication, n (%) | 454 (18.6) | 238 (28.2) | 135 (17.2) | 23 (21.5) | <0.001 |
| C-D Grade ≥3, n (%) | 221 (9.0) | 117 (13.8) | 80 (10.2) | 12 (11.2) | <0.001 |
| EJ leak (TG/PG only), n (%) | - | 14 (1.7) | - | 1 (0.9) | NA |
| Duodenal stump leak (TG/DG only), n (%) | 14 (0.6) | 7 (0.8) | - | - | NA |
| GJ/GD/GG anastomosis leak (DG/PPG/PG), n (%) | 33 (1.4) | - | 10 (1.3) | 0 | NA |
| Fluid collection, n (%) | 98 (4.0) | 65 (7.7) | 25 (3.2) | 7 (6.5) | <0.001 |
| 30-day mortality, n (%) | 2 (0.1) | 1 (0.1) | 1(0.1) | 0 | 0.964 |
| Pre-NLR | 2.02 ± 1.18 | 2.27 ± 1.50 | 1.97 ± 1.21 | 2.04 ± 0.97 | <0.001 |
| Early NLR | 8.74 ± 4.93 | 10.17 ± 6.54 | 7.90 ± 5.75 | 8.66 ± 4.72 | <0.001 |
| Late NLR | 1.62 ± 1.24 | 1.76 ± 4.20 | 1.60 ±1.26 | 1.48 ±0.87 | 0.364 |

DG = Distal Gastrectomy; TG = Total Gastrectomy; PPG = Pylorus-Preserving Gastrectomy; PG = Proximal Gastrectomy; C-D = Clavien-Dindo; EJ = Esophagojejunostomy; GJ = Gastrojejunostomy; GD = Gastroduodenostomy; GG = Gastrogastrostomy
